# Supplementary material for: Comparative effectiveness and cost-effectiveness of three first-line EGFR-tyrosine kinase inhibitors: Analysis of real-world data in a tertiary hospital in Taiwan
Source: PLoS One. 2020 Apr 8;15(4):e0231413. doi: 10.1371/journal.pone.0231413 (PMC7141611; doi:10.1371/journal.pone.0231413)
Supplement: S1 Appendix — (DOC) [file pone.0231413.s001.doc]

**Comparative effectiveness and cost-effectiveness of three first-line EGFR-tyrosine kinase inhibitors: Analysis of real-world data in a tertiary hospital in Taiwan**

Szu-Chun Yang, Wu-Wei Lai, Jason C. Hsu, Wu-Chou Su, Jung-Der Wang

Corresponding author: Jung-Der Wang, [jdwang121@gmail.com](mailto:jdwang121@gmail.com)

**Fig A.** Propensity-score matching the subjects for analysis. EGFR: epidermal growth factor receptor; (EQ-5D) EuroQol five-dimension questionnaire; NSCLC: non-small cell lung cancer; QoL: quality-of-life measurement; TKI: tyrosine kinase inhibitor.

**Fig B.** Progression-free survival and overall survival by different first-line treatments. *P* value using log-rank test for curve comparison.

**Fig C.** Mean QoL curves using kernel smoothing (i.e., moving averages of the nearby 10% values). Each “x” denotes a QoL measurement at the specific time after treatment. QoL: quality of life.

**Fig D.** Lifetime psychometric scores in 2 domains and 4 facets after different first-line treatments.

| **Table A.** T790 mutation and costs of subsequent osimertinib. | | | | |
| --- | --- | --- | --- | --- |
|  | Median  PFS | Erlotinib  *n* = 48 | Afatinib  *n* = 48 | Gefitinib  *n* = 96 |
| Serum/tissue T790M, *n* (%) |  |  |  |  |
| Positive | 9.7 months [1] | 9 (18.8%) | 6 (12.5%) | 14 (14.6%) |
| Negative | 2.8 months [1] | 5 (10.4%) | 9 (18.8%) | 8 (8.3%) |
| Not applicable |  | 34 (70.8%) | 33 (68.8%) | 74 (77.1%) |
| Subsequent use of osimertinib, *n* (%) |  | 9 (18.8%) | 9 (18.8%) | 16 (16.7%) |
| Cumulative use of osimertinib, months |  | 87.3 | 66.6 | 141.4 |
| Monthly costs of osimertinib, US$ |  | 6,030 | | |
| Additional costs per person, US$ |  | 10,968 | 8,367 | 8,883 |
| Adjusted ICER |  |  |  |  |
| △Cost /△Life expectancy, US$/life year |  | 17,767 | dominated | -- |
| △Cost /△QALE, US$/QALY |  | 26,650 | dominated | -- |
| ICER: incremental cost-effectiveness ratio; PFS: progression-free survival; QALE: quality-adjusted life expectancy; QALY: quality-adjusted life year; T790M: substitute mutation of threonine (T) with methionine (M) at position 790 of exon 20 | | | | |

**Reference:**

1. Oxnard GR, Thress KS, Alden RS, Lawrance R, Paweletz CP, Cantarini M, et al. Association between plasma genotyping and outcomes of treatment with osimertinib (AZD9291) in advanced non-small-cell lung cancer*. J Clin Onc*ol. 2016;34(28):3375-82. Epub 2016/06/30. doi: 10.1200/jco.2016.66.7162. PubMed PMID: 27354477; PubMed Central PMCID: PMCPMC5035123.
