# Supplementary figures and images for: Comparative effectiveness and cost-effectiveness of three first-line EGFR-tyrosine kinase inhibitors: Analysis of real-world data in a tertiary hospital in Taiwan
Source: PLoS One. 2020 Apr 8;15(4):e0231413. doi: 10.1371/journal.pone.0231413 (PMC7141611; doi:10.1371/journal.pone.0231413)

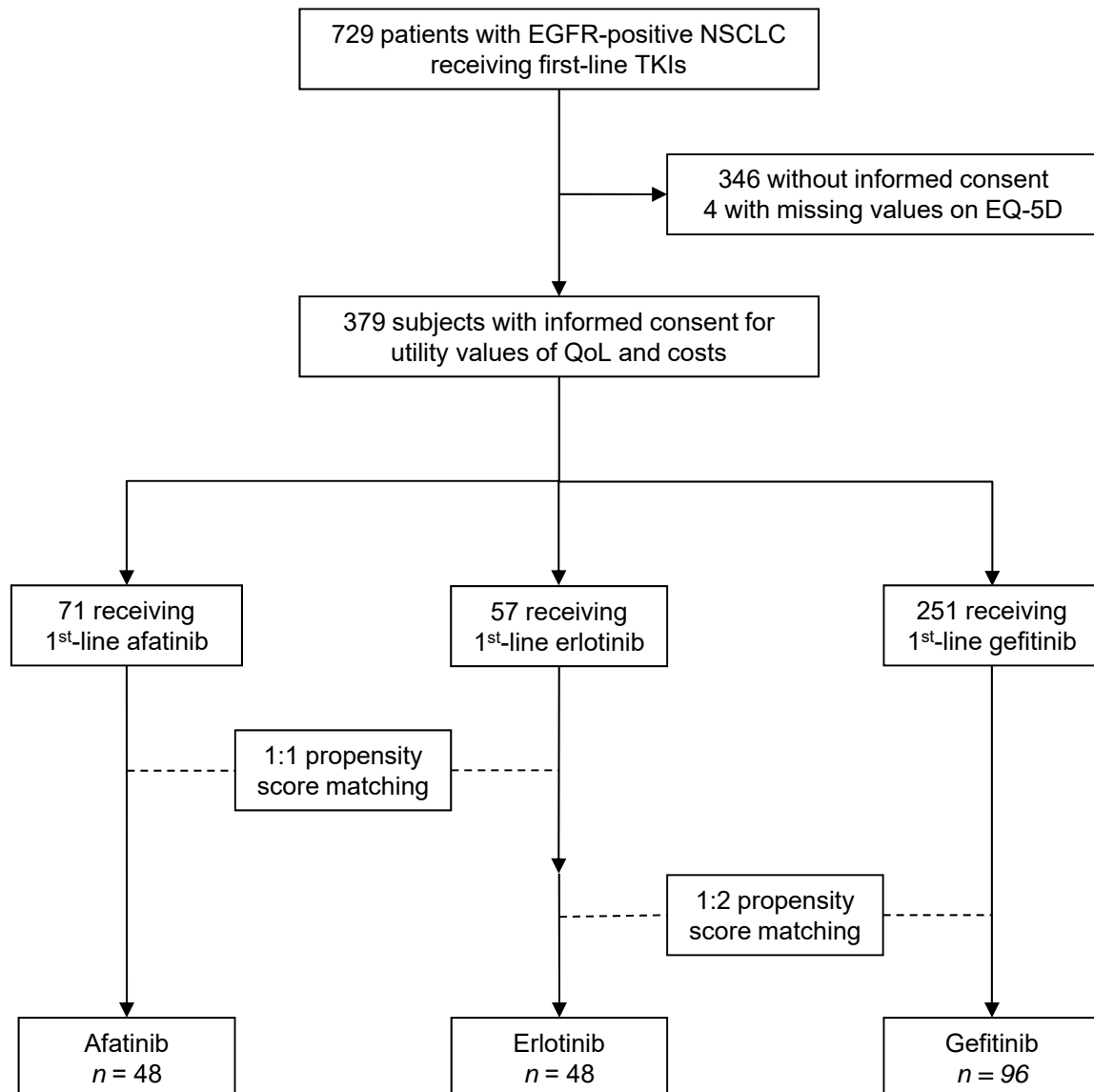

**Figure A**

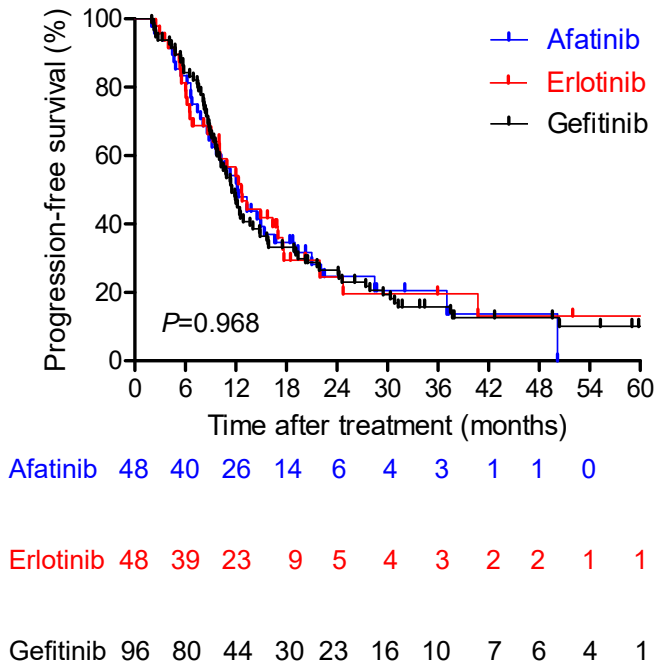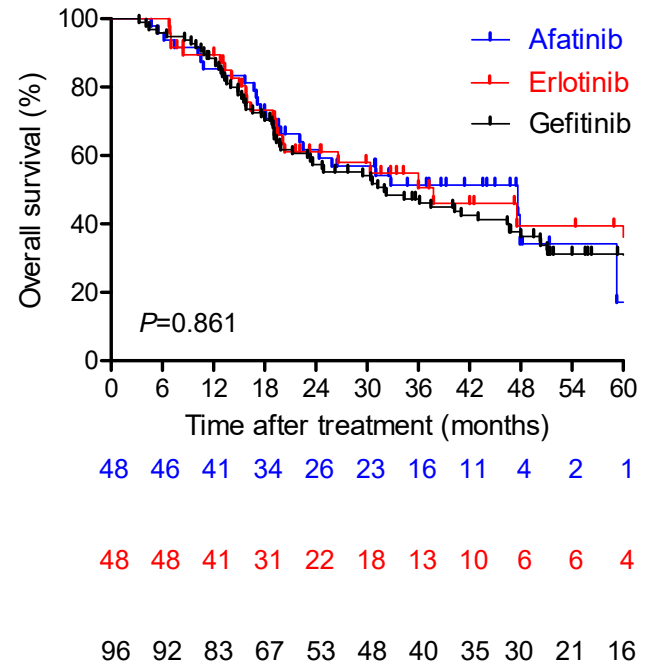

**Figure B**

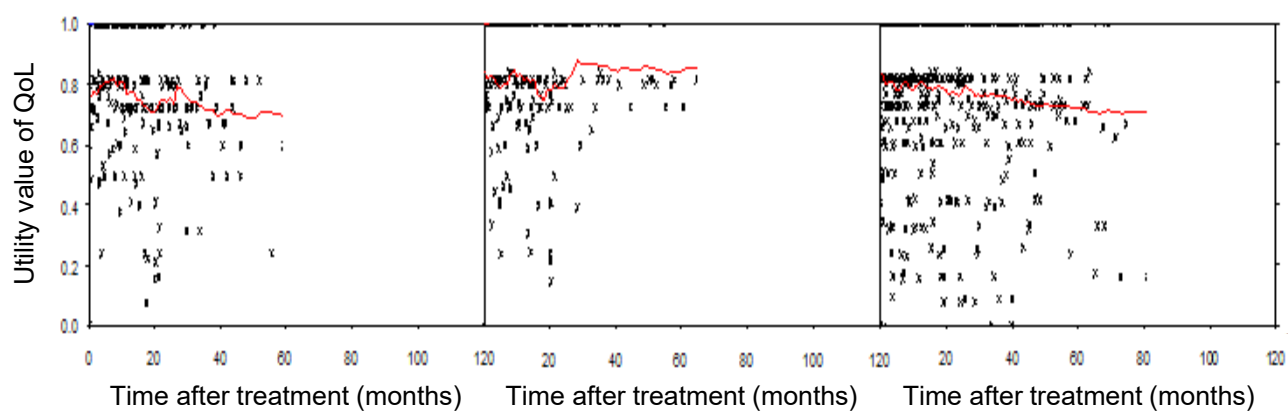

**Figure C**

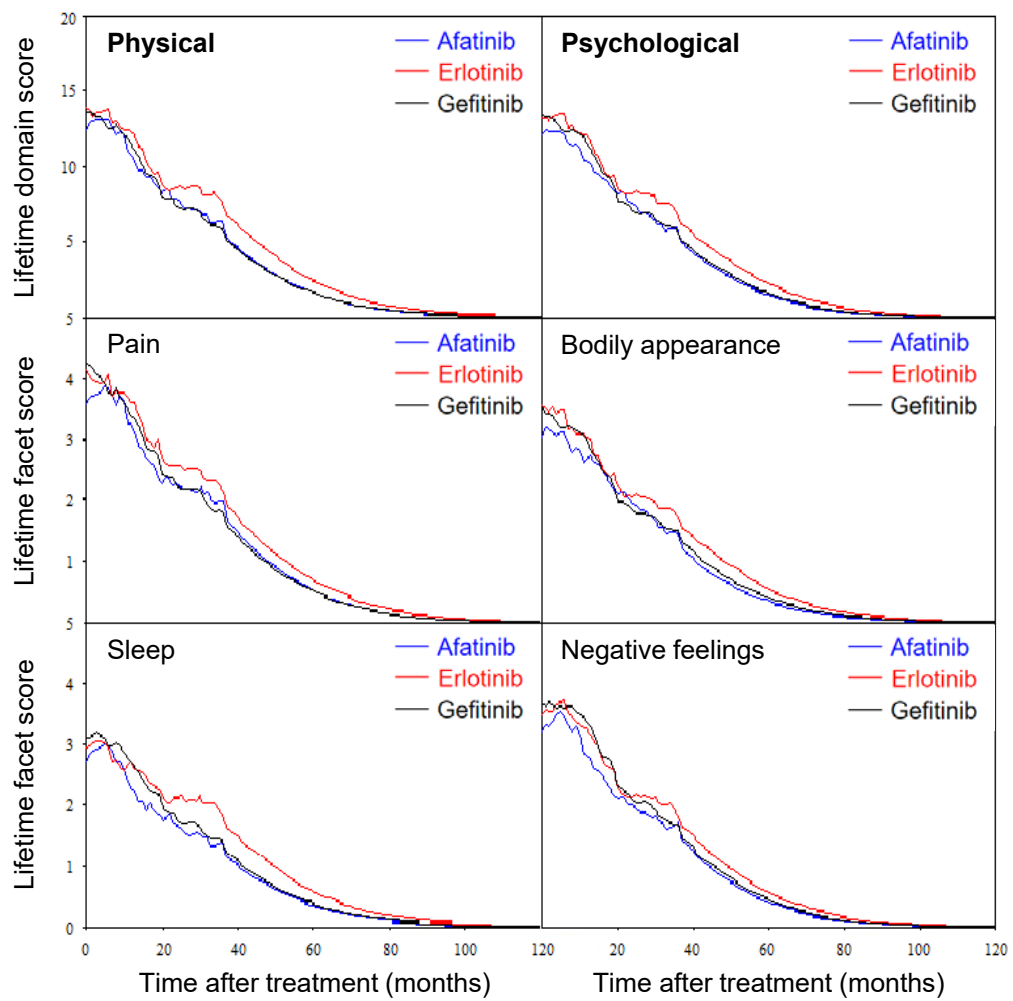

**Figure D**

Supplement: S1 File — (PDF) [file pone.0231413.s003.pdf]
