# Supplementary material for: Comparative effectiveness and cost-effectiveness of three first-line EGFR-tyrosine kinase inhibitors: Analysis of real-world data in a tertiary hospital in Taiwan
Source: PLoS One. 2020 Apr 8;15(4):e0231413. doi: 10.1371/journal.pone.0231413 (PMC7141611; doi:10.1371/journal.pone.0231413)
Supplement: Attachment — Submitted filename: Response to Reviewers.doc [file pone.0231413.s005.doc]

**Editor:**

**Comment 1 Please add Cost-effectiveness Planes (Figures) using your ICERs.**

**Response 1** Thank you for the comment! We have added cost-effectiveness scatter plots **(Fig. 2)** in the **Results** section. Because the probabilistic sensitivity analysis compared three treatments at the same time, we followed Reviewer #2’s advice and plotted the scatters of them by using lifetime costs and life expectancy (QALE). We did not specify any EGFR-tyrosine kinase inhibitor (TKI) as the comparator and used the incremental values.

**(Page 12, line 6)**

Fig. 2 shows the cost-effectiveness scatter plots.

**(Figure 2)**

Fig 2. Cost-effectiveness scatter plots using (A) life expectancy and (B) QALE. Individual dots represent results after 1,000 iterations. QALE: quality-adjusted life expectancy; QALY: quality-adjusted life year.

**Comment 2 Please add ICER results in your abstract.**

**Response 2** Thank you for the comment! Compared with the gefitinib group, afatinib was dominated by erlotinib. Therefore, we directly showed the ICERs of erlotinib, which were US$17,960/life year and US$12,782/QALY. The results have been added to the **Abstract**.

**(Abstract: results)**

Considering 3 treatments together, afatinib was dominated by erlotinib. Erlotinib had an incremental cost-effectiveness of US$17,960/life year and US$12,782/QALY compared with gefitinib.

**Comment 3 Please also interpret ICERs in your results section.**

**Response 3** Thank you for the comment! We have added ICER results in the **Results** section and interpreted them in the **Discussion** section.

**(Page 12, lines 2-4)**

The erlotinib group dominated the afatinib group and had an incremental cost-effectiveness of US$17,960/life year and US$12,782/QALY when compared with the gefitinib group.

**(Page 13, line 15)**

We thus tentatively concluded that erlotinib appeared to be cost-effective (Table 2).

**(Page 15, lines 17,18)**

Erlotinib appeared to be cost-effective from payer’s perspective.

**Reviewer #1:**

**Comment 1 Lack of generalizability must be addressed in Discussion and you need to change the Title to honestly indicate that this work is done in a single tertiary hospital in Taiwan.**

**Response 1** Thank you for the comment! We have modified the **Title** and addressed this issue in the **Introduction** and **Discussion** sections.

**(Title)**

Comparative effectiveness and cost-effectiveness of three first-line EGFR-tyrosine kinase inhibitors: Analysis of real-world data in a tertiary hospital in Taiwan

**(Page 6, line 4)**

Using the new method and real-world data of a tertiary hospital in Taiwan, this study attempted to directly compare the effectiveness and cost-effectiveness of three first-line EGFR-TKIs.

**(Page 14, lines 18,19)**

Several limitations must be acknowledged in this study. First, the work was done in a tertiary hospital in Taiwan, generalizing the results must be cautious.

**Comment 2 Methods has to be fully revised as there is lack of information. Especially, what information other than QOL and survival are collected prospectively for this research purpose. If you are utilizing the information from EMR which was collected as a part of routine care, it is fine but you need to mention it and how those additional information are abstracted from EMR. It might be helpful to just attach the study protocol approved by IRB before commencement as a supplemental document if you wish to avoid re-writing Method entirely.**

**Response 2** Thank you for the comment! We created a system to abstract age, sex, performance and recurrence statuses at the initiation of therapy from electrical medical records. Because all these data are required to be approved for receiving the first-line EGFR-TKIs in our hospital, the information collected were relatively complete, leaving few patients with missing performance statuses. In addition, we reviewed the reports of brain magnetic resonance imaging and computed tomography with contrast to define brain metastasis. That is, subjects who did not receive brain images or show any radiographic evidence at the initiation of therapy were categorized as negative for brain metastasis. In addition, a thoracic oncologist independently reviewed every chest computed tomography, bone scan, positron emission tomography, and brain image to determine if there is any disease progression. Disease progression was defined according to the Response Evaluation Criteria in Solid Tumors (RECIST) version 1.1. We have revised the **Methods** section extensively to report the details.

**(Page 7, lines 2-9)**

We created a system to abstract age, sex, performance and recurrence statuses at the initiation of therapy from electrical medical records. Because all these data are required to be approved for receiving the first-line EGFR-TKIs in our hospital, the information collected were relatively complete, leaving few patients with missing performance statuses. In addition, we reviewed the reports of brain magnetic resonance imaging and computed tomography with contrast to define brain metastasis. That is, subjects who did not receive brain images or show any radiographic evidence at the initiation of therapy were categorized as negative for brain metastasis.

**(Page 8, lines 6-8)**

A thoracic oncologist independently reviewed every chest computed tomography, bone scan, positron emission tomography, and brain image to determine if there is any disease progression. Disease progression was defined according to the Response Evaluation Criteria in Solid Tumors (RECIST) version 1.1 [18].

**Comment 3 Information is missing for how you handled missing data in QOL or data abstracted from other source which needs to be implemented in either body or supplemental document. Moreover, information about how you summarized QOL data in Figure 1 while every patient has at different timing of visit in real-world setting need to be included.**

**Response 3** Thank you for the comment! Patients with missing values (i.e., ineffective for utility calculation) on EQ-5D questionnaires were initially excluded in the analysis. We have modified it in the **Methods** and **Results** sections, as well as **Fig. A** in S1 Appendix. Because age, sex, performance and recurrence statuses are required to be approved for receiving the first-line EGFR-TKIs in our hospital, only 12 patients showed missing performance statuses. We also reviewed images of every patient to determine if brain metastasis and disease progression were present, these data were complete. Missing numbers of patients without performance status have been listed on the **Table 1**.

**(Page 6, lines 17,18)**

There were 729 patients with EGFR mutation-positive advanced NSCLC under first-line TKIs during the study period. After excluding patients without informed consent and cases with missing values on EuroQol five-dimension (EQ-5D) questionnaires, all subjects were included for analysis.

**(Page 10, lines 22,23)**

From May 2011 to December 2017, a total of 729 patients in NCKUH received afatinib, erlotinib, or gefitinib as first-line therapies for EGFR mutation-positive advanced NSCLC. Among them, 346 cases did not sign the informed consent and 4 cases with missing values on EQ-5D questionnaires, leaving 379 subjects (Fig. A in S1 Appendix).

**(Fig. A in S1 Appendix, upper part)**

**(Table 1)**

**Response 3 (continued)** The time after treatment for each QoL measurement was defined as the period between the initiation of treatment and that of the interview. We used kernel-smoothing (i.e., a moving average of the nearby 10%) to estimate the mean QoL function after initiation of treatment. The QoL scores beyond the follow-up period were assumed to be the same as the average of the last 10% near the end of follow-up. We multiplied lifetime survival function by the mean QoL function to obtain quality-adjusted survival curve, with the sum of the areas under the curve being the QALE. We have addressed this issue in the **Methods** and **Results** sections and added a **Fig. C** in S1 Appendix for developing mean QoL curves using kernel smoothing.

**(Page 9, lines 5-12)**

The time after treatment for each QoL measurement was defined as the period between the initiation of treatment and that of the interview. We used kernel-smoothing (i.e., a moving average of the nearby 10%) to estimate the mean QoL function after initiation of treatment [22]. The QoL scores beyond the follow-up period were assumed to be the same as the average of the last 10% near the end of follow-up. We multiplied lifetime survival function by the mean QoL functions of EQ-5D and WHOQOL to obtain quality-adjusted survival curves, with the sum of the areas under the curves being the QALE and lifetime psychometric scores, respectively.

**(References)**

22.	Hwang JS, Wang JD. Integrating health profile with survival for quality of life assessment. *Qual Life Res*. 2004;13(1):1-10; discussion 1-4.

**(Page 11, lines 16,17)**

Fig. 1 depicts the cost- and quality-adjusted survival curves along time courses for different treatments. In this figure, the survival probability was multiplied by the costs and QoL at each time point *t* (see Fig. C in S1 Appendix for the mean QoL curve using moving averages of the nearby 10% values), the sums of the shaded areas under the curves represent the lifetime costs and QALE, respectively.

**(Fig. C in S1 Appendix)**

Fig C. Mean QoL curves using kernel smoothing (i.e., moving averages of the nearby 10% values). Each “x” denotes a QoL measurement at the specific time after treatment. QoL: quality of life.

**Comment 4 In section of Effectiveness, it is written that the authors determined disease progression based on RECIST v1.1. I wonder if this is true because disease progression is not strictly assessed based on RECIST criteria but also by other evidence such as patient symptoms in real-world setting. Some may decide the switching of therapy due to adverse event too. If the authors wish to mention that the authors reevaluated disease progression by independent review, there needs to be clarification.**

**Response 4** Thank you for the comment! A thoracic oncologist independently reviewed every chest computed tomography, bone scan, positron emission tomography, and brain image to determine if there is any disease progression. A chest tomography is mandatory for applications of EGFR-TKIs or subsequent therapies every 3 months. Switching of therapy due to adverse events without an image progression was not considered as disease progression. We have clarified the issue in the **Methods** section.

**(Page 8, lines 6-11)**

A thoracic oncologist independently reviewed every chest computed tomography, bone scan, positron emission tomography, and brain image to determine if there is any disease progression. Disease progression was defined according to the Response Evaluation Criteria in Solid Tumors (RECIST) version 1.1 [18]. Switching of therapy due to adverse events without an image progression was not considered as disease progression.

**Comment 5 Please make a change to supplemental figure 1 in order to specify how you identify 379 patients from 729 patients overall. you need to clarify the reason of exclusion as it is not clear by just mentioning the detailed information of QOL and cost was provided by 379 patients. (what do you mean by the detailed information?)**

**Response 5** Thank you for the comment! There were 729 patients with EGFR mutation-positive advanced NSCLC under first-line TKIs during the study period. Among them, 346 cases did not sign the informed consent and 4 cases with missing values on EQ-5D questionnaires, leaving 379 in the analysis set. We have made corrections to the **Methods**, **Results**, and **Fig. A** in S1 Appendix.

**(Page 6, lines 15-19)**

There were 729 patients with EGFR mutation-positive advanced NSCLC under first-line TKIs during the study period. After excluding patients without informed consent and cases with missing values on EuroQol five-dimension (EQ-5D) questionnaires, all subjects were included for analysis.

**(Page 10, lines 22,23)**

From May 2011 to December 2017, a total of 729 patients in NCKUH received afatinib, erlotinib, or gefitinib as first-line therapies for EGFR mutation-positive advanced NSCLC. Among them, 346 cases did not sign the informed consent and 4 cases with missing values on EQ-5D questionnaires, leaving 379 subjects (Fig. A in S1 Appendix).

**(Fig. A in S1 Appendix)**

Please kindly refer to the response of Comment 3.

**Comment 6 Table 1 has to be explained in detail why these clinical characteristics are chosen for propensity score matching. Smoking status is well known prognostic factor in NSCLC. If information was not collected, you have to mention in Discussion that not all known prognostic factors are adjusted in this research. Moreover, clarification is needed for why these certain comorbidities are selected even though there are several other comorbidities which may affect QOL of NSCLC patient treated with EGFR-TKI such as skin rash.**

**Response 6** Thank you for the comment! Previous literatures have found performance status, recurrence status, metastasis, and mutation subtypes to be prognostic factors of survival and QoL outcomes among patients receiving first-line EGFR-TKIs. Therefore, we computed propensity scores by age, sex, and these clinical characteristics upon initiation of treatment. We have explained it in the **Methods** section. The comorbidities we initially showed on Table 1 were based on discharge diagnoses (i.e., diagnoses of hospitalization). However, since none of them was chosen for propensity score matching, we have omitted the comorbidities in Table 1 to avoid confusion. Unobserved prognostic factors including smoking were not considered because of the lack of data. We have acknowledged it as one of the limitations in **Discussion** section. However, most of EGFR mutation-positive NSCLC patients in Asia (including Taiwan) are never smokers, the results would not be biased too much.

**(Page 7, lines 15-19)**

Previous literatures have found performance status, recurrence status, metastasis, and mutation subtypes to be prognostic factors of survival [11] and QoL outcomes [12] among patients receiving first-line EGFR-TKIs. Therefore, we computed propensity scores by age, sex, and these clinical characteristics upon initiation of treatment.

**(References)**

11.	Okamoto I, Morita S, Tashiro N, Imamura F, Inoue A, Seto T, et al. Real world treatment and outcomes in EGFR mutation-positive non-small cell lung cancer: Long-term follow-up of a large patient cohort. *Lung Cancer*. 2018;117:14-9.

12.	Yang SC, Lin CC, Lai WW, Chang SM, Hwang JS, Su WC, et al. Dynamic changes in quality of life after three first-line therapies for EGFR mutation-positive advanced non-small-cell lung cancer. *Ther Adv Med Oncol.* 2018;10:1758834018755072.

**(Page 15, lines 5-9)**

Several limitations must be acknowledged in this study. … Third, unobserved prognostic factors including smoking were not considered into propensity score matching because of the lack of data. However, most of EGFR mutation-positive NSCLC patients in Asia (including Taiwan) are never smokers [1], the results would not be biased too much.

**(References)**

1.	Yatabe Y, Kerr KM, Utomo A, Rajadurai P, Tran VK, Du X, et al. EGFR mutation testing practices within the Asia Pacific region: results of a multicenter diagnostic survey. *J Thorac Oncol*. 2015;10(3):438-45.

**Comment 7 It is better to mention each drug costs in Taiwan as Table 2 only show the total healthcare expenditure as you mention afatinib is too expensive in Discussion. Moreover, availability of generic drugs for gefitinib or erlotinib in Taiwan can be included in Discussion.**

**Response 7** Thank you for the comment! We have added the cost of each drug in the **Results** and **Table 1**. During the study period (2011-2017), generic drug of gefitinib was not available and its efficacy was not disclosed in Taiwan. If the price of afatinib was reduced 25% to match that of gefitinib, it would not be strongly dominated (an ICER of US$42,455/life year). We have mentioned this issue in the **Discussion** section.

**(Page 11, lines 2,3)**

**Table 1** shows the 192 propensity score-matched patients by treatment as well as those without matching or excluded. The daily prices per person were US$48.2, US$37.0, and US$36.3 for 40mg afatinib, 150mg erlotinib, and 250mg gefitinib, respectively.

**(Table 1)**

**(Page 13, lines 19-22)**

That is, the costs of EGFR-TKIs were a major determinant of cost-effectiveness. During the study period of 2011-2017, generic drugs of gefitinib were not yet available in Taiwan. If the price of afatinib had been reduced 25% to match that of gefitinib (Table 1), it would not have been strongly dominated.

**Comment 8 Figure 1 is key result for this research, therefore, need to be explained in details. Especially, there seems sudden increase in cost and QOL for afatinib and erlotinib. Also, there may be an over prediction of QoL when reaching to survival probability close to 0.**

**Response 8** Thank you for the comment! Medical costs usually drop after initiation of therapies but increase in final months due to end-of-life care. Because it is difficult to measure QoL scores close to death, we hypothesized the values beyond the follow-up period to be the same as that near the end of follow-up, which might lead to an over-estimation of QALE. We must admit that numbers of subjects in the afatinib and erlotinib groups were smaller than those receiving gefitinib. Consequently, mean QoL scores and costs after 2 years of follow-up were more easily influenced by outliers. However, as the survival rates after 2 or more years would be low, the bias would not be too big to affect the inference. We have revised the **Results** and addressed the limitation in **Discussion** section.

**(Page 11, lines 18-20)**

**Fig. 1** depicts the cost- and quality-adjusted survival curves along time courses for different treatments. … As expected, costs dropped after initiation of therapies but increased in final months due to end-of-life care [24].

**(References)**

24.	Hwang JS, Hu TH, Lee LJ, Wang JD. Estimating lifetime medical costs from censored claims data. *Health Econ*. 2017;26(12):e332-e44.

**(Page 15, lines 9-15)**

Several limitations must be acknowledged in this study. … Fourth, because it is difficult to measure QoL scores close to death, we hypothesized the values beyond the follow-up period to be the same as that near the end of follow-up, which might lead to an over-estimation of QALE. Moreover, numbers of subjects in the afatinib and erlotinib groups were smaller than those receiving gefitinib. Consequently, mean QoL scores and costs after 2 years of follow-up were more easily influenced by outliers. However, as the survival rates after 2 or more years would be low, the bias would not be too big to affect the inference.

**Reviewer #2:**

**Comment 1 The research was innovative on methodology in two approaches. First, it used a semi-parametric approach to extrapolate the overall survival (OS) over lifetime. Second, the study patients were matched individually on propensity scores.**

**The authors claimed the study was based on the real-world dataset of Taiwanese patients that compared treatment effectiveness directly across three TKIs. Regarding the first approach, the OS relied on a statistical model of data projection as the study patients were not followed exhaustively through lifetime. The study justified this approach adequately by citing previous studies for validity of the employed model.**

**Response 1** Thank you for the appreciation and kind words! The extrapolation method has been shown to be effective via computer simulations, mathematical proof, and corroboration by examples of lung cancer cohorts.

**Comment 2 Regarding the second approach, comparability across patients which was based on the propensity-score matching (PSM) across the three TKI groups have not been adequately addressed. To make the three groups comparable, the PSM handles selection bias through the observed covariates. This was justified through balances in the selected 7 covariates shown in Table 1. The PSM, however, could not control for the unobserved prognostic factors which can lead to omitted-variable bias. An instrumental-variable (IV) technique is a viable alternative to randomization and probably is better than PSM. First, the study should provide adequate argument on how well PSM performed as compared to meta-analysis (direct and indirect treatment comparisons) based on RCTs and pseudo-randomization technique like IV.**

**Response 2** Thank you for the comment! To account for observed covariates among three different EGFR-TKIs, we used propensity score matching. Previous literatures have found performance status, recurrence status, metastasis, and mutation subtypes to be prognostic factors of survival and QoL outcomes among patients receiving first-line EGFR-TKIs. Therefore, we computed propensity scores by age, sex, and these clinical characteristics upon initiation of treatment. The balances between afatinib, erlotinib, and gefitinib were tested using standardized differences, an absolute value less than 0.1 suggests each two groups are well balanced. However, given small numbers of the subjects, we must admit that some of the covariates were not well balanced. For example, the afatinib group had a lower proportion of patients with brain metastases. But it would not threaten the conclusion that erlotinib appeared to be cost-effective. Although instrumental variables can control unobserved covariates, such a variable may not easily be found in all study settings. Meta-analysis pooling randomized trials data can avoid confounding, but the inclusion and exclusion criteria may limit its application in every day practice. Thus, our method matching each group with propensity scores based on real-world data could still be a viable alternative in providing useful information for allocation of limited resources. We have revised the **Methods**, **Results**, and **Discussion** sections, as well as **Table 1**.

**(Page 7, lines 10 and 15-21)**

To account for observed covariates among three different EGFR-TKIs, we used propensity score matching. … Previous literatures have found performance status, recurrence status, metastasis, and mutation subtypes to be prognostic factors of survival [11] and QoL outcomes [12] among patients receiving first-line EGFR-TKIs. Therefore, we computed propensity scores by age, sex, and these clinical characteristics upon initiation of treatment. The balances between afatinib, erlotinib, and gefitinib were tested using standardized differences, an absolute value less than 0.1 suggests each two groups are well balanced.

**(References)**

11.	Okamoto I, Morita S, Tashiro N, Imamura F, Inoue A, Seto T, et al. Real world treatment and outcomes in EGFR mutation-positive non-small cell lung cancer: Long-term follow-up of a large patient cohort. *Lung Cancer*. 2018;117:14-9.

12.	Yang SC, Lin CC, Lai WW, Chang SM, Hwang JS, Su WC, et al. Dynamic changes in quality of life after three first-line therapies for EGFR mutation-positive advanced non-small-cell lung cancer. *Ther Adv Med Oncol.* 2018;10:1758834018755072.

**(Table 1)**

**(Page 11, lines 8,9)**

After propensity score matching, most of the characteristics in the three groups were balanced.

**(Page 13, lines 8-14)**

Although instrumental variables can control unobserved covariates [35], such a variable may not easily be found in all study settings. Meta-analysis pooling randomized trials data can avoid confounding [36], but the inclusion and exclusion criteria may limit its application in every day practice. Thus, our method matching each group with propensity scores based on real-world data could still be a viable alternative in providing useful information for allocation of limited resources. We thus tentatively concluded that erlotinib appeared to be cost-effective (Table 2).

**(References)**

35.	Crown WH. Propensity-score matching in economic analyses: comparison with regression models, instrumental variables, residual inclusion, differences-in-differences, and decomposition methods. *Appl Health Econ Health Policy*. 2014;12(1):7-18.

36.	Holleman MS, Al MJ, Zaim R, Groen HJM, Uyl-de Groot CA. Cost-effectiveness analysis of the first-line EGFR-TKIs in patients with non-small cell lung cancer harbouring EGFR mutations. *Eur J Health Econ*. 2019. doi: 10.1007/s10198-019-01117-3. [Epub ahead of print].

**Comment 3 The second issue for the second approach is on the study-excluded and included patients. There were 729 patients in total that received the first-line TKIs. Then 350 patients were excluded due to data availability. The remaining 379 patients (52% of 729) were subject to the PSM, where only another 50% (192/379 patients) were successfully matched. The study should shed some lights on the excluded 537 patients in total (74% of 729) that were eligible but did not contribute the analytic dataset.**

**Response 3** Thank you for the comment! We categorized the patients into 192 propensity-score matched patients, 187 excluded patients after matching, and 350 patients excluded in the beginning because of lack of informed consent (*n* = 346) or with missing values (*n* = 4). Clinical characteristics of each group have been summarized in **Table 1**. In general, propensity-score matched patients had higher proportions of men, brain metastasis, and common mutations compared with those without matching or excluded in the beginning. Moreover, they were younger, showed a better performance status, and had a better progression-free survival than patients excluded in the beginning. We have added some sentences in the **Results** section and modified the title of **Table 3**.

**(Page 10, line 25 to Page 11, line 8)**

**Table 1** shows the 192 propensity score-matched patients by treatment as well as those excluded in the beginning and after matching. … In general, propensity-score matched patients had higher proportions of men, brain metastasis, and common mutations compared with those without matching or excluded in the beginning. Moreover, they were younger, showed a better performance status, and had a better progression-free survival than patients excluded in the beginning.

**(Table 1)**

**(Page 12, lines 12,13)**

We also examined the overall results of the 379 patients before propensity score matching (Table 3).

**(Table 3)**

Table 3. Costs, effectiveness, and ICER of the 379 patients before propensity score matching.

**Comment 4 Page 3: “… would improve patients’ values.” The term “patients’ values” is ambiguous; the values on which exact aspects should be pointed out: quality of life, clinical benefits, values for money, or else? This should be more specific.**

**Response 4** Thank you for the comment! It would improve patients’ clinical benefits and save costs. We have changed “patients’ values” to these words.

**(Abstract: introduction)**

Comparison of the effectiveness and cost-effectiveness of three first-line EGFR-tyrosine kinase inhibitors (TKIs) would improve patients’ clinical benefits and save costs.

**Comment 5 Page 5: “Randomized trials have shown similar efficacy of these three agents [3, 4].” Reference #3 which was not relevant should not be cited in the present study since it was conducted in patients mixed between 1st line and 2nd line treatments. Reference #4 showed a statistically significant difference in the efficacy measured by PFS, in which afatinib was better than gefitinib (despite non-significant difference in OS because the study was not mature yet).**

**Response 5** Thank you for the comment! Following your advice, we have deleted the reference (#3 in the previous version of manuscript). A randomized trial showed afatinib is superior to first-generation TKIs in progression-free survival; however, a significant difference in overall survival has not been revealed. We have revised in the **Introduction** section.

**(Page 5, lines 6-9)**

Although a randomized trial showed afatinib is superior to first-generation TKIs in progression-free survival [3], a significant difference in overall survival has not been revealed [4].

**(References)**

3.	Park K, Tan EH, O'Byrne K, Zhang L, Boyer M, Mok T, et al. Afatinib versus gefitinib as first-line treatment of patients with EGFR mutation-positive non-small-cell lung cancer (LUX-Lung 7): a phase 2B, open-label, randomised controlled trial. *Lancet Oncol*. 2016;17(5):577-89.

4.	Paz-Ares L, Tan EH, O'Byrne K, Zhang L, Hirsh V, Boyer M, et al. Afatinib versus gefitinib in patients with EGFR mutation-positive advanced non-small-cell lung cancer: overall survival data from the phase IIb LUX-Lung 7 trial. *Ann Oncol*. 2017;28(2):270-7.

**Comment 6 Page 5: “To improve patients’ values, the comparative effectiveness …” The term “patients’ values” again is ambiguous. How the patients’ values could be improved by exploration of the comparative effectiveness and cost-effectiveness of TKIs was unclear?**

**Response 6** Thank you for the comment! We have omitted the ambiguous words and used “to improve patients’ clinical benefits and save costs” in the **Introduction** section.

**(Page 5, lines 10,11)**

To improve patients’ clinical benefits and save costs, the comparative effectiveness and cost-effectiveness of these drugs warrant further exploration.

**Comment 7 Page 5: “When the cost-effectiveness is considered from the payers’ perspective, real-world data seem more credible.” Credibility on the baseline survivals or credibility on the comparative effectiveness and cost-effectiveness? The real-world data based on local population tend to be useful for generating the baseline outcomes for the comparator which is least effective such as placebo or chemotherapy. When comparing across innovative drugs (TKIs), the data should be based on the “randomized, controlled” studies, the level-1 evidence. Please provide the references on the evidence that real-world data are more credible from the payer’s perspective.**

**Response 7** Thank you for the comment! We have deleted the word “credible” and lowered our tone in the **Introduction** section. A cost-effectiveness study using real-world approach would be useful in assisting healthcare resources allocation.

**(Page 5, lines 19,20)**

Although trial data are generally cleaner, their restrictive inclusion and exclusion criteria and limited length of follow-up period may limit the application in daily practice. A cost-effectiveness study using real-world approach would be useful in assisting healthcare resources allocation.

**Comment 8 Page 6: “This study was approved by the International Review Board of …” This is typo? It should say the Institutional Review Board of …**

**Response 8** Thank you for the comment! We have corrected the typo in the **Methods** section.

**(Page 6, line 8)**

This study was approved by the Institutional Review Board of National Cheng Kung University Hospital (NCKUH) before commencement (A-ER-100-079).

**Comment 9 Page 6: “Among 1,828 enrolled cases, 379 patients with EGFR mutation-positive advanced NSCLC under first-line TKIs were abstracted for analysis.” There were 350 patients not included in the study (Supplementary Table 1, the rightmost column) and 379 patients had detailed information of QoL and costs (shown in Supplementary Figure 1), of which 192 patients were successfully matched. The total of 729 (379+350) patients were relevant to the study and should be mentioned as the enrolled cases instead of all invited lung cancer cases (1,828 patients), which some of them did not receive the EGFR-TKIs.**

**Otherwise, Supplementary Figure 1 should show the patient flow diagram beginning with a total of 1,828 patients invited, followed by those 1,099 patients who did not receive TKIs were excluded, then 350 patients without information were excluded and 379 remained in the dataset.**

**Response 9** Thank you for the comment! There were 729 patients with EGFR mutation-positive advanced NSCLC under first-line TKIs during the study period. We excluded 346 patients without informed consent and 4 cases with missing values on EQ-5D questionnaires, leaving 379 subjects. After 1:1:2 (afatinib: erlotinib: gefitinib) propensity-score matching the patients, 192 patients were used for analysis. We have modified **Fig. A** in S1 Appendix beginning with 729 patients. The **Methods** and **Results** sections have also been revised.

**(Page 6, lines 15-19)**

There were 729 patients with EGFR mutation-positive advanced NSCLC under first-line TKIs during the study period. After excluding patients without informed consent and cases with missing values on EuroQol five-dimension (EQ-5D) questionnaires, all subjects were included for analysis.

**(Page 10, lines 20-25)**

From May 2011 to December 2017, a total of 729 patients in NCKUH received afatinib, erlotinib, or gefitinib as first-line therapies for EGFR mutation-positive advanced NSCLC. Among them, 346 cases did not sign the informed consent and 4 cases with missing values on EQ-5D questionnaires, leaving 379 subjects (Fig. A in S1 Appendix). After 1:1:2 (afatinib: erlotinib: gefitinib) propensity-score matching the patients, 192 patients were used for analysis.

**(Fig. A in S1 Appendix)**

**Comment 10 The study should elaborate on (1) what were the reasons they were excluded? (2) How did the excluded patients look like and were they much different in characteristics from those included in the study?**

**To answer (2), the excluded 537 patients in total (in Table 1) should be divided further into two columns separately between 350 patients first excluded (as in Supplementary Table 1) and 187 patients without PSM.**

**Response 10** Thank you for the comment! We excluded 346 patients without informed consent and 4 cases with missing values on EQ-5D questionnaires. We have revised the **Methods**, **Results**, and **Fig. A** in S1 Appendix. Following your advice, we have also modified the **Table 1** to show the clinical characteristics of 192 propensity score-matched patients, 187 patients without matching, and 350 patients excluded in the beginning.

**(Page 6, lines 17-19)**

There were 729 patients with EGFR mutation-positive advanced NSCLC under first-line TKIs during the study period. After excluding patients without informed consent and cases with missing values on EuroQol five-dimension (EQ-5D) questionnaires, all subjects were included for analysis.

**(Page 10, lines 22,23)**

From May 2011 to December 2017, a total of 729 patients in NCKUH received afatinib, erlotinib, or gefitinib as first-line therapies for EGFR mutation-positive advanced NSCLC. Among them, 346 cases did not sign the informed consent and 4 cases with missing values on EQ-5D questionnaires, leaving 379 subjects (Fig. A in S1 Appendix). After 1:1:2 (afatinib: erlotinib: gefitinib) propensity-score matching the patients, 192 patients were used for analysis.

**(Fig. A in S1 Appendix)**

Please kindly refer to the response of Comment 9.

**(Page 10, line 25 to Page 11, line 6)**

**Table 1** shows the 192 propensity score-matched patients stratified by treatments as well as those excluded in the beginning and after matching. … In general, propensity-score matched patients had higher proportions of men, brain metastasis, and common mutations compared with those without matching or excluded in the beginning. Moreover, they were younger, showed a better performance status, and had a better progression-free survival than patients excluded in the beginning.

**(Table 1)**

**Comment 11 Page 6” “To minimize selection bias and ensure better comparability among three different EGFR-TKIs, we used propensity score matching via a greedy algorithm.”**

**The term “selection bias” should be replaced with more specific terms that the propensity score matching (PSM) aims for and can handle. For example, the PSM is only able accounted for the observed covariates that have been used for determining the probability of obtaining a treatment. However, it is not able to handle selection bias due to the “omitted variables”.**

**The term “greedy algorithm” should be elaborated technically. For example, how is this method different from conventional approaches in calculating the propensity scores, such as probit or logistic regressions? Does the algorithm perform well for the not so large sample size (379 patients in this study).**

**Response 11** Thank you for the comment! We omitted the term “selection bias.” Propensity score matching was used to account for observed covariates among three different EGFR-TKIs. Actually, the propensity score of each group was computed using logistic regression. “Greedy algorithm” means that the first treated unit was selected to find its closest control based on the difference of their propensity scores. This procedure was repeated for all the treated units. We have revised the paragraph in **Methods** section.

**(Page 7, lines 10-13)**

To account for observed covariates among three different EGFR-TKIs, we used propensity score matching via greedy algorithm [10]. That is, the first treated unit was selected to find its closest control based on the difference of their propensity scores using logistic regression. The procedure was repeated for all the treated units.

**(References)**

10. Coca-Perraillon M. Local and global optimal propensity score matching. SAS Global Forum. 2007;185. Available from: <https://support.sas.com/resources/papers/proceedings/proceedings/forum2007/185-2007>

**Comment 12 Page 7: As the research used both EQ-5D and WHOQOL-BREF for QoL measures, it should be point out which specific measure was used for adjusting the survival years. What are the reasons to analyze the lifetime QoL scores if they were not used for estimating QALE or QALYs.**

**Otherwise, the study should propose in the beginning the lifetime QoL scores as one of the objectives, say the patients’ values.**

**Response 12** Thank you for the comment! By integrating the long-term survival with utility values of QoL, we estimated the quality-adjusted life expectancy (QALE). Because psychometric scores are more sensitive than the utility values and may provide additional information for effectiveness evaluation, we also multiplied survival by the WHOQOL scores for lifetime psychometric scores. We have revised the **Introduction** and **Methods** sections.

**(Page 5, line 24 to Page 6, line 3)**

By integrating the long-term survival with utility values of QoL and costs, we developed a method to estimate the quality-adjusted life expectancy (QALE) and lifetime costs. Because psychometric scores are more sensitive than the utility values [8] and may provide additional information for effectiveness evaluation, lifetime psychometric scores were also estimated.

**(References)**

8.	Yang SC, Kuo CW, Lai WW, Lin CC, Su WC, Chang SM, et al. Dynamic changes of health utility in lung cancer patients receiving different treatments: A 7-year follow-up. *J Thorac Oncol*. 2019;14(11):1892-900.

**(Page 9, lines 10-12)**

We multiplied lifetime survival function by the mean QoL functions of EQ-5D and WHOQOL to obtain quality-adjusted survival curves, with the sum of the areas under the curves being the QALE and lifetime psychometric scores, respectively.

**Comment 13 Page 8: Data on the medical costs were collected until December 2017 for calculating monthly expenditures per case. However, the censor date for survival was in September 2018. Were the medical costs occurring in patients survived during December 2017 to September 2018 not accounted for?**

**Response 13** Thank you for the comment! Under IRB’s approval, the medical costs were available until December 2017. Similar to the estimation of QALE, costs beyond the follow-up period were assumed to be the same as the average of the last 10% near the end of follow-up. That is, we still accounted for the medical costs incurring between December 2017 and September 2018. We have added a sentence in the **Methods** section.

**(Page 9, lines 22-24)**

Specifically, the total monthly healthcare expenditures were divided by the effective sample sizes, i.e., the number of patients who survived in that month, to obtain the average monthly healthcare expenditures per case. Similar to the estimation of QALE, costs beyond the follow-up period were assumed to be the same as the average of the last 10% near the end of follow-up. These values were subsequently multiplied by the corresponding monthly survival probabilities and summed to obtain the lifetime costs per case.

**Comment 14 Since costs of the TKIs per se played an important role, especially for afatinib in the lifetime costs as shown in the study results. The data on the unit price of each TKI along with the descriptive statistics of utilities per TKI should be shown as the baseline parameters.**

**Response 14** Thank you for the comment! We have added the information in the **Results** and **Table 1**. The daily prices per person were US$48.2, US$37.0, and US$36.3 for 40mg afatinib, 150mg erlotinib, and 250mg gefitinib, respectively. Patients receiving erlotinib had a higher mean utility value compared with the afatinib and gefitinib groups. However, to present the utility value for each group, we constructed linear mixed models to consider random effects from subjects because EQ-5Ds were repeatedly measured. The intercept represents the mean utility value. We have addressed it in the **Methods** section.

**(Page 8, lines 20-22)**

To present the utility value for each group, we constructed linear mixed models to consider random effects from subjects because EQ-5Ds were repeatedly measured. The intercept represents the mean utility value.

**(Page 11, lines 2,3 and 10-12)**

Table 1 shows the 192 propensity score-matched patients stratified by treatment as well as those excluded in the beginning and after matching. The daily prices per person were US$48.2, US$37.0, and US$36.3 for 40mg afatinib, 150mg erlotinib, and 250mg gefitinib, respectively. … Patients receiving erlotinib had a higher mean utility value compared with the afatinib and gefitinib groups.

**(Table 1)**

**Comment 15 Page 9: “We assumed a gamma distribution for life expectancy, QALE, and costs, …” As healthcare costs are known for very skewed distribution, assuming a gamma distribution of cost data is well accepted. However, a gamma distribution for life expectancy, QALE is not familiar with. References for justifying the distribution of LE and QALE would be helpful.**

**Response 15** Thank you for the comment! Although a previous article (*Value Health* 2013; 16:922-931) hypothesized a gamma distribution for life expectancy and QALE, we recognized the distributions are not very skewed. Therefore, we assumed a normal distribution for them and re-conducted the sensitivity analyses. Changes in the **Abstract**, **Methods**, **Results**, and **Figure 3** have been made.

**(Abstract: results)**

Acceptability curves showed that erlotinib had 58.6% and 78.9% probabilities being cost-effective given a threshold of 1 Taiwanese per capita GDP per life year and QALY, respectively.

**(Page 10, line 9)**

We assumed a normal distribution for life expectancy and QALE [23], and a gamma distribution for costs, with means and standard deviations set to base-case values.

**(References)**

23.	Michael D, Mark JS, Karl C, Stoddart GL, George WT. Methods for the economic evaluation of health care programmes. 4th ed. Oxford, United Kingdom: Oxford University Press; 2015.

**(Page 12, lines 6-11)**

A Monte Carlo simulation with 1,000 iterations was conducted to construct the acceptability curves (**Fig. 3A**), which show erlotinib had a probability of 58.6% being cost-effective at a cost-effectiveness threshold of US$24,408 (1 GDP per capita of Taiwan in 2017) / life year. If the willingness-to-pay threshold was set at US$24,408 / QALY (**Fig. 3B**), the probability became 78.9%.

**(Figure 3)**

**Fig 3.** Acceptability curves of cost-effectiveness thresholds using (A) US$/life year, and (B) US$/QALY. The dash line represents a threshold of US$24,408 (1 GDP per capita of Taiwan in 2017) / life year or QALY. GDP: gross domestic product; QALY: quality-adjusted life year.

**Comment 16 Page 9: “A Monte Carlo micro-simulation with 1,000 iterations was conducted to construct acceptability curves…” A Monte Carlo micro-simulation is too general to be mentioned for the PSA. There were three TKI options to be compared at the same time on cost-effectiveness measures. Specific approaches, either net monetary benefit or net health benefit used for determining the most cost-effective option among the three should be mentioned instead.**

**Response 16** Thank you for the comment! To determine the most cost-effective option using net life years or QALY gained, a Monte Carlo simulation was conducted. We have mentioned it in the **Methods** section.

**(Page 10, lines 10,11)**

To determine the most cost-effective option using net life years or QALY gained, a Monte Carlo simulation with 1,000 iterations was conducted to construct acceptability curves of the three different EGFR-TKIs.

**Comment 17 The study should present descriptive statistics in the beginning of the Results on the unit price of each TKI along with the utilities per TKI as the baseline parameters.**

**Response 17** Thank you for the comment! The daily prices per person were US$48.2, US$37.0, and US$36.3 for 40mg afatinib, 150mg erlotinib, and 250mg gefitinib, respectively. Patients receiving erlotinib had a higher mean utility value compared with the afatinib and gefitinib groups. We have added the information in the **Results** and **Table 1**.

**(Page 11, lines 2,3 and 10-12)**

**Table 1** shows the 192 propensity score-matched patients stratified by treatment as well as those excluded in the beginning and after matching. The daily prices per person were US$48.2, US$37.0, and US$36.3 for 40mg afatinib, 150mg erlotinib, and 250mg gefitinib, respectively. … Patients receiving erlotinib had a higher mean utility value compared with the afatinib and gefitinib groups.

**(Table 1)**

Please kindly refer to the response of Comment 14.

**Comment 18 Table 2: The results on CER should be omitted as the “average ratio” has no implications for policy decision and can create confusions if they were contradicted to the incremental analysis.**

**Instead, the results on ICER should be calculated hierarchically either by an increasing order in the total costs or by an increasing order in the total effectiveness (LYs, QALE, QALYs).**

**Based on an increasing order of QALYs, the first ICER should be calculated for afatinib (vs. gefitinib). As erlotinib was more effective and less costly than afatinib, there was no need to calculate the ICER for erlotinib (vs. afatinib) because erlotinib was cost-saving in lay language or technically economic dominance. In this case, it should not say afatinib was least cost-effective. Instead, it should say afatinib was not cost effective since it was just simply dominated by erlotinib and should be dropped totally for the cost-effectiveness comparison.**

**Then, the ICER for erlotinib (vs. gefitinib) could be calculated and compared with Taiwan’s GDP per capita. In this case, the ICER of erlotinib (vs. gefitinib) was below Taiwanese GDP per capita, hence, the study should conclude that erlotinib was cost effective.**

**Response 18** Thank you for the constructive comment! Following your kind advice, we have deleted the results on CER. We calculated the ICERs hierarchically by an increasing order in life expectancy (QALE). The changes have been made in **Table 2** and **Table 3**. The erlotinib group dominated the afatinib group and had an incremental cost-effectiveness of US$17,960/life year and US$12,782/QALY when compared with the gefitinib group. We tentatively concluded that erlotinib appeared to be cost-effective. Other changes have also been shown in the **Abstract**, **Results**, and **Discussion** sections.

**(Abstract: results)**

Considering 3 treatments together, afatinib was dominated by erlotinib. Erlotinib had an incremental cost-effectiveness of US$17,960/life year and US$12,782/QALY compared with gefitinib. Acceptability curves showed that erlotinib had 58.6% and 78.9% probabilities of being cost-effective given a threshold of 1 Taiwanese per capita GDP per life year and QALY, respectively.

**(Abstract: conclusions)**

Erlotinib appeared to be cost-effective.

**(Page 12, lines 2-4)**

Costs, effectiveness, and ICER of the 192 propensity score-matched patients are summarized in Table 2. … The erlotinib group dominated the afatinib group and had an incremental cost-effectiveness of US$17,960/life year and US$12,782/QALY when compared with the gefitinib group.

**(Page 13, line 15)**

We thus tentatively concluded that erlotinib appeared to be cost-effective (Table 2).

**(Page 15, lines 17,18)**

Erlotinib appeared to be cost-effective from payer’s perspective.

**(Table 2)**

**(Table 3)**

**Comment 19 Page 11: Since the PSA compared all three TKIs mutually at the same time, Supplementary Figure 4 should not do a pairwise-plot. Instead, it should plot the scatters for all three TKIs in the same graph by using total cost and total QALYs of each TKI rather than using the incremental values by specifying any TKI as the comparator. In addition, there is no need to draw the CE threshold of Taiwan at this stage.**

**Response 19** Thank you for the comment! We omitted pairwise figures and plotted the scatters for all three EGFR-TKIs using lifetime costs and life expectancy (QALE). We also did not show the willingness-to-pay threshold. Following Editor’s comment, we have shifted the supplementary figure to **Figure 2**.

**(Page 10, line 16)**

Cost-effectiveness scatter plots were also developed.

**(Page 12, line 6)**

**Fig. 2** shows the cost-effectiveness scatter plots.

**(Figure 2)**

**Fig 2.** Cost-effectiveness scatter plots using (A) life expectancy and (B) QALE. Individual dots represent results after 1,000 iterations. QALE: quality-adjusted life expectancy; QALY: quality-adjusted life year.
