# Supplementary material for: Comparative effectiveness and cost-effectiveness of three first-line EGFR-tyrosine kinase inhibitors: Analysis of real-world data in a tertiary hospital in Taiwan
Source: PLoS One. 2020 Apr 8;15(4):e0231413. doi: 10.1371/journal.pone.0231413 (PMC7141611; doi:10.1371/journal.pone.0231413)
Supplement: Attachment — Submitted filename: Response to Reviewers_1.doc [file pone.0231413.s006.doc]

**Reviewer #1:**

**Comment 1 As for my previous comment 5, you included the detailed explanation regarding the reason why 350 patients were excluded from this research in the manuscript and supplemental figure 1. If the reason for exclusion of 346 out of 350 patients is no informed consent obtain for this research, then it is not appropriate to use their data and summarize in Table 1 from the ethical point of view. Please delete all relevant data from patients without signed informed consent in this manuscript.**

**Response 1** Thank you for the comment! Following your kind advice, we have omitted the whole column in **Table 1** and relevant words in the **Results** section. Moreover, their data in the **S2 Appendix** (Supporting Information) have also been deleted.

**(Page 11, lines 1,2)**

**Table 1** shows the 192 propensity score-matched patients stratified by treatment as well as those excluded ~~in the beginning and~~ after matching.

**(Page 11, lines 5-8)**

In general, propensity-score matched patients had higher proportions of men, brain metastasis, and common mutations compared with those without matching ~~or excluded in the beginning~~. ~~Moreover, they were younger, showed a better performance status, and had a better progression-free survival than patients excluded in the beginning.~~

**(Table 1)**

| **Table 1.** Clinical characteristics of the 192 propensity score-matched patients and those excluded after matching. | | | | | | |
| --- | --- | --- | --- | --- | --- | --- |
|  | Propensity score-matched patients  *n* = 192 | | | | | Excluded patients  after matching |
|  | Afatinib (A) | Erlotinib (E) | Gefitinib (G) | Standardized differences | |  |
|  | *n* = 48 | *n* = 48 | *n* = 96 | A vs. E | G vs. E | *n* = 187 |
| Daily price per person, US$ | 48.2 | 37.0 | 36.3 |  | |  |
| Age, *n* (%) | | | | | | |
| < 67 years | 32 (66.7) | 31 (64.6) | 60 (62.5) | 0.04 | -0.04 | 120 (64.2) |
| ≥ 67 years | 16 (33.3) | 17 (35.4) | 36 (37.5) | -0.04 | 0.04 | 67 (35.8) |
| Male, *n* (%) | 21 (43.8) | 21 (43.8) | 39 (40.6) | 0 | -0.06 | 63 (33.7) |
| Mutation subtype, *n* (%) | | | | | | |
| Exon 19 deletions | 24 (50.0) | 23 (47.9) | 46 (47.9) | 0.04 | 0 | 70 (37.4) |
| L858R substitution | 22 (45.8) | 24 (50.0) | 49 (51.0) | -0.08 | 0.02 | 91 (48.7) |
| Other mutations | 2 (4.2) | 1 (2.1) | 1 (1.0) | 0.12 | -0.08 | 26 (13.9) |
| Performance status, *n* (%) | | | | | | |
| ECOG: 0-1 | 45 (93.8) | 45 (93.8) | 85 (88.5) | 0 | -0.18 | 168 (89.8) |
| ECOG: 2-4 | 3 (6.3) | 3 (6.3) | 11 (11.5) | 0 | 0.18 | 18 (9.6) |
| Missing | 0 | 0 | 0 |  |  | 1 (0.5) |
| Disease by recurrence, *n* (%) | | | | | | |
| Recurrent | 9 (18.8) | 8 (16.7) | 12 (12.5) | 0.06 | -0.11 | 45 (24.1) |
| Newly-diagnosed | 39 (81.3) | 40 (83.3) | 84 (87.5) | -0.06 | 0.11 | 142 (75.9) |
| Brain metastasis, *n* (%) | 20 (41.7) | 25 (52.1) | 52 (54.2) | -0.21 | 0.04 | 14 (7.5) |
| PFS, median (IQR) months | 12.3 (7.1-22.2) | 12.7 (6.4-22.0) | 11.5 (8.2-24.3) |  | | 12.1 (6.7-23.8) |
| Number of QoLs, *n* | 200 | 194 | 491 |  | | 951 |
| Utility value, *β_0_* (SE)^a^ | 0.80 (0.02) | 0.85 (0.02) | 0.81 (0.02) |  | | 0.82 (0.01) |
| ^a^Intercept of linear mixed model considering random effects from subjects. ECOG: Eastern Cooperative Oncology Group; PFS: progression-free survival | | | | | | |
